# Association between a Tetranucleotide Repeat Polymorphism of *SPAG16* Gene and Cataract in Male Children

**DOI:** 10.1155/2013/810395

**Published:** 2013-09-27

**Authors:** Shipra Mehra, Suman Kapur, Suma Ganesh

**Affiliations:** ^1^School of Public Health and Psychiatry Institute, University of Illinois, 1601 West Taylor Street, 306N Chicago, IL 60612, USA; ^2^Biological Sciences Department, Birla Institute of Technology and Science Pilani, Hyderabad Campus, Jawahar Nagar, Shameerpet Mandal, R. R. District, Hyderabad 500078, India; ^3^Department of Paediatric Ophthalmology and Strabismus, Dr. Shroff's Charity Eye Hospital, 5027 Kedar Nath Road, Daryaganj, New Delhi 110 002, India

## Abstract

*Purpose*. Studies involving genotyping of STR markers at 2q34 have repeatedly found the region to host the disease haplotype for pediatric cataract. Present study investigated the association of D2S2944 marker, in sperm associated antigen 16 (*SPAG16)* gene and rs2289917 polymorphism, in *γ*-crystallin B gene, with childhood cataract. *Methods*. 97 pediatric cataract cases and 110 children with no ocular defects were examined for tetranucleotide repeat marker/SNP using PCR-SSLP/RFLP techniques. Polymorphisms were assessed for association using contingency tables and linkage disequilibrium among alleles of the markers was estimated. Energy-optimization program predicted the secondary structure models of repeats of D2S2944. *Results*. Seven alleles of D2S2944, with 9–15 “GATA” repeats, were observed. Frequency of the longer allele of D2S2944, ≥(GATA)_13_ repeats, was 0.73 in cases and 0.56 in controls (*P* = 0.0123). Male children bearing ≥(GATA)_13_ repeats showed >3-fold higher risk for cataract (CI_95%_ = 1.43–7.00, *P* = 0.0043, *P*_*c*_ = 0.0086) as compared to female children (OR = 1.19, CI_95%_ = 0.49–2.92, *P* = 0.70). Cases with haplotype—≥(GATA)_13_ of D2S2944 and “C” allele rs2289917—have a higher risk for pediatric cataract (OR = 2.952, CI_95%_ = 1.595~5.463, *P* = 0.000453). >(GATA)_13 _ repeats formed energetically more favorable stem-loop structure. *Conclusion*. Intragenic microsatellite repeat expansion in *SPAG16* gene increases predisposition to pediatric cataract by probably interfering posttranscriptional events and affecting the expression of adjacent lens transparency gene/s in a gender bias manner.

## 1. Introduction

Cataract is a major cause of treatable childhood blindness, with a prevalence of around 5 to 15 cases per 10,000 children in India [1]. Cataract in children is particularly serious because it has the potential for inhibiting visual development, resulting in permanent blindness and disability. Inherited cataracts represent 8–25% of infantile cataract cases [2]. Understanding the genetics of cataract will not only lead to better treatment approaches but also open avenues for effective counseling. Most inherited cataracts mapped on to chromosome 2 are associated with a subgroup of genes, namely, gamma-crystallins (*CRYG*) present at 2q33–35, encoding proteins important for maintenance of lens transparency and homeostasis [3]. The chromosomal region from 198 Mb to 220 Mb on chromosome 2q has been repeatedly found to host the disease haplotype for pediatric cataract [4]. Infact Cat-Map database summary shows that major portion of mutations/variations observed to be associated with cataracts in this region have been reported in Asians majorly including Indians and Chinese.

**Figure 1:**
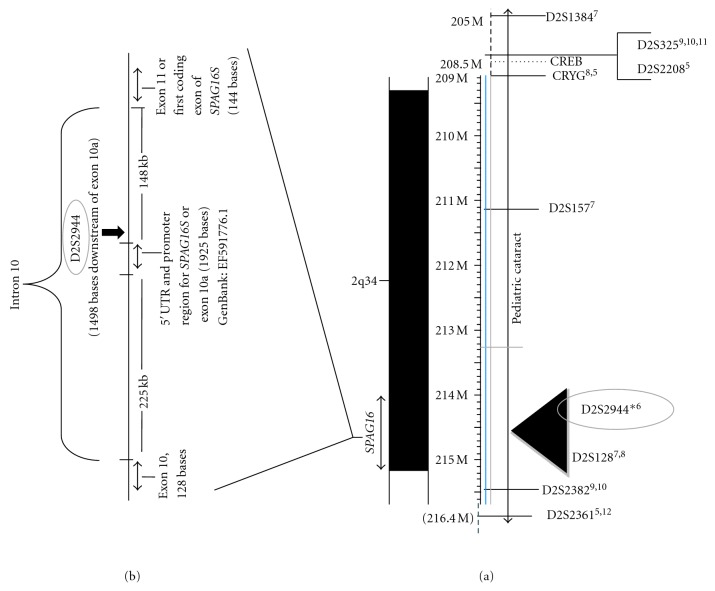
Cytogenetic localization at 2q34 of markers associated with cataract and location of D2S2944 marker in *SPAG16* gene. (a) known markers in the region flanked by D2S1384 and D2S2361 are shown. The ruler in the partial map of chromosome 2 shows the relative nucleotide position expressed in mega bases (205Mb–215 Mb) and all the markers distances are according to NCBI: Mapviewer. Marshfield shows markers D2S2944, D2S2361, D2S128 at genetic map position 210.43cM. Alleles of the microsatellite marked in figure are reported to form the disease haplotype linked to congenital/pediatric cataract. (b) From the NCBI Reference Sequence: NW_001838863.1 showing 420.70kb region from base 39766685 to 40187388. Superscripts correspond to references: Nandrot et al., (2003) [5], Iyengar et al., (2004) [6], Mackay et al., (2004) [7], Rogaev et al., (1996) [8], Stephan et al. (1999) [9], Shentu et al., (2004) [10], Li et al., (2008) [11], and Zhang et al., (2009) [12].

Our preliminary cataract case-control analyses of four microsatellite markers namely D2S1384, D2S2944, D2S2359 and D2S439, within the region 2q33-37 revealed significant association with D2S2944—an intragenic polymorphic marker located in intron 10 of the Sperm Associated Antigen 16 gene, *SPAG16* (NT_005403.17) at chromosome 2q34 (Figure 1(b)). Many investigators have used this marker, at 210.43 cM for genotyping familial cohorts [5–7] and have reported the region 2q34 to be in linkage with cataract [8–12] (Figure 1(a)). Moreover, microdeletions of the region 2q32–34 are known to be accompanied with vision defects and genital anomalies in boys [13]. Another gene in this region, *γ*-crystallin B gene (*CRYGB*), has rs2289917—a tagged single nucleotide polymorphism (SNP). Studies in our lab showed it to be significantly associated with pediatric cataract [4] and further analysis pointed towards a gender bias association. Male children with the variant (“C”) allele have a >4-fold risk for developing cataract (OR = 4.30, CI_95%_ = 2.07–8.94, *P* < 0.0001) as compared to female children with no risk (OR = 1.31, CI_95%_ = 0.39–4.38, *P* = 0.75). Other known polymorphisms studied at *γ*-crystallin locus like exonic SNP's of *CRYGA* (T196C-Exon 3) and *CRYGB* (G449T-Exon 2) as well as the noncoding SNP in *CRYGA* (G198A-Intron A) differed insignificantly between the cataract cases and controls in our pediatric cohort.

Since linkage studies cite marker/s cosegregating with large genomic regions, usually multiple Mb in size and including many genes, it becomes imperative to resolve the region by fine mapping. A marker consistently falling under the disease linked haplotype or region showing peak linkage signal in several studies points towards a gene/s in vicinity associated with the disease etiology. To our knowledge, till date, genes in the 2q34 region have not been tested for their association with cataract and the gender specific effects observed for this locus. The location of this intragenic marker-D2S2944 suggested that *SPAG16* gene may have a role in pathophysiology of lens opacification as several studies have demonstrated that microsatellites in the non-coding region may function in gene regulation [14]. SPAG16 protein exists in two isoforms, the L and the S. While the *Spag16L* mRNA has been detected in testis, brain, lung, oviduct and other murine tissues containing cells with a “9 + 2” axoneme structure, *Spag16S* is only expressed in testis or male germ cell in mice [15]. Spag16 S being a bifunctional protein, on one hand interacts with MEIG1 (meiosis expressed gene 1 product involved in chromosome/chromatin-binding and participates in the regulation of chromosome structure and/or gene expression) and on the other, acts as a transcription factor (TF) that transactivates the promoter of the L isoform [16]. Functionally, the L isoform is responsible for axoneme stability and sperm flagellar motility [16–18]. Mice chimeric for a mutation deleting the transcripts for both SPAG16L and SPAG16S have a profound defect in spermatogenesis [15]. We hypothesized that the D2S2944 microsatellite may act as an enhancer or repressor to regulate *SPAG16* gene expression which in turn impacts gene/s regulating lens transparency in gender specific manner. In this study, we investigated the association between variations of the (GATA)_*n*_ repeats in microsatellite marker D2S2944 in the* SPAG16* gene and a tagged SNP-rs2289917, in the promoter of the *γ*-crystallin Bgene, with childhood cataract.

## 2. Material and Method

All participants in this study were recruited after obtaining a written informed consent. The patient cohort included unrelated pediatric patients who attended Dr. Shroff's Eye Hospital for cataract surgery. The type of cataract was recorded according to the morphological classification proposed by Merin [19]. Patients with uveitis, cataract due to trauma, steroid therapy or infective etiology, cataract with associated glaucoma or retinal pathology or subluxated lens and patients' positive for TORCH were excluded from the present study. The control population comprised of children with both lenses graded as having no opacities on observation under slit lamp and no history of congenital/infantile, juvenile, traumatic/postsurgical cataract or any other detectable ocular defects. Controls were drawn from the same ethnic population as patients from the same geographical region (subjects residing in and around Malka Ganj to Darya Ganj in Delhi). The study was conducted following the norms of Declaration of Helsinki for human experimentation and was approved by the Institutional Human Ethics Committee (IHEC) of both BITS and the eye hospital. Genomic DNA was obtained from healthy children and children with cataract using method described elsewhere [20]. Genomic DNA was subjected to PCR using UniSTS primer set (http://www.ncbi.nlm.nih.gov/genome/sts/sts.cgi?uid=68648/), and the number of (GATA)_*n*_ repeats were determined by resolving PCR amplicons in 12% nondenaturing polyacrylamide gel electrophoresis with commercial and internal in-house standard DNA ladders as described by Mehra et al. 2012 [21]. rs2289917 was genotyped by PCR-RFLP as reported earlier [20]. Two independent observers assigned the genotypes and unambiguous genotypes were assigned to 97 cases and 110 controls. Chi square test (*χ* ^2^), trend test, Fisher's exact test and Odds ratios (OR) with 95% confidence interval (CI_95%_) were used to test differences between the cases and controls and *P* values were Bonferroni corrected (*P*_*c*_), wherever applicable. All statistics were performed using Med Calc version 9.3.9.0. To elucidate the mechanism by which the microsatellite motif could be involved in regulating the *SPAG16* expression, the potential secondary structure/s in the intron 10 of *SPAG16A *mRNA were predicted using the authentic and minimum free energy (ΔG*; *kcal/mol) method of MFOLD program (version 3.1) (http://mfold.rit.albany.edu/?q=mfold/RNA-Folding-Form/). Haplotype frequencies for pairs of alleles of D2S2944 and rs2289917, as well as *χ* ^2^ values for allele associations, and linkage disequilibrium (LD) coefficients *D*′ were estimated by SHEsis software (http://analysis2.bio-x.cn/myAnalysis.php/).

## 3. Result 

**Figure 2:**
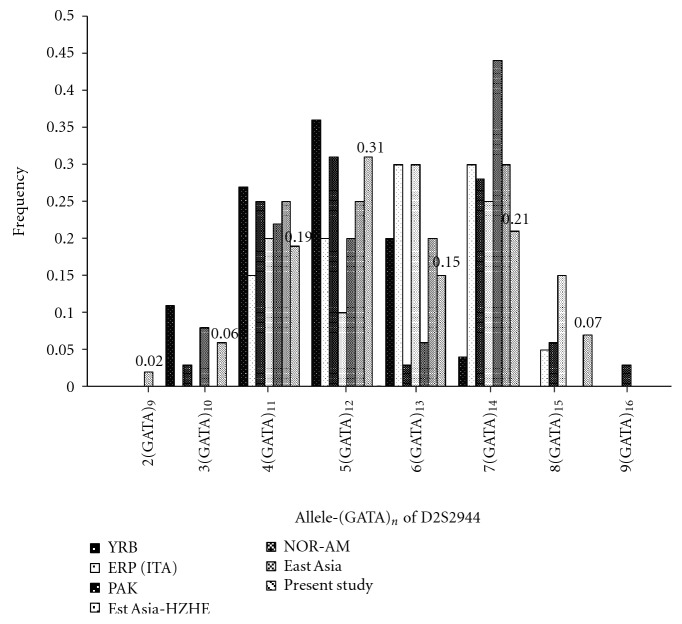
Allele distribution of D2S2944: across various populations of the world. YRB: Yourba Africa; ERP (ITA): Italian; PAK: Pakistan; EST ASIA-HZHE: Hezhen from north east China; NOR-AM:Pima Mexico; EAST ASIA: Uigur from Turpan county China; present study: North Indians (controls only).

Of the 110 control subjects,mean age 5.43 ± 3.6 years, 59% were males and remaining 41% were females. Similarly, among 97 patients with cataract, mean age 5.23 ± 3.9 years, 55% were males and 45% were females. 90% cases were simplex/sporadic cataract cases with majority of them having bilateral cataract (80%). Only 10% of the recruited cases reported a positive family history for cataract. Following the Merin criteria of classification [19], zonular cataract (lamellar, nuclear, oil droplet, cortical, coronary sutural, pulverulent, cerulean, or coralliform) was observed in 47% of eyes examined, followed by total (31%, includes mature, complete, morgagnian, or disk-like) and polar (14%, includes anterior polar, anterior pyramidal, anterior subcapsular, anterior lenticonus, posterior subcapsular, posterior lenticonus, or posterior cortical) opacities. Membranous cataract (capsular) was seen in mere 5% of the recruited patient cohort and the rest 3% of cases had cataract associated with preexistent posterior capsule defect and with Pulfrich Phenomenon. Population data (ALFRFED: http://alfred.med.yale.edu/alfred/keywordsearchRes.asp and TPMD: http://tpmd.nhri.org.tw/php-bin/index_en.php database) showed that D2S2944 is a highly polymorphic STR, heterozygosity of 0.77, with nine alleles yielding an amplicon of 100–132 bp, corresponding to 8–16 (GATA)_*n*_ tetra-nucleotide repeats. In this cohort of 207 Asian Indian subjects, a total of seven alleles were observed, namely allele 2–8, corresponding to 9–15 (GATA)_n_ nucleotide repeats. The most frequently observed allele in controls was allele 5 (31%) followed by allele 7 (21%), allele 4 (19%) and allele 6 (15%), Table 1. Observed allele frequencies along with the world frequencies are depicted in Figure 2. The system is in Hardy-Weinberg equilibrium and the Exact test [22] gave a *P* value of >0.05. 

As can be seen from Figure 2, there are differences in individual allele frequency within the East Asian populations. The most frequent D2S2944 allele in Europeans is allele 6 and 7 (13 and 14 “GATA” repeats), whereas allele 5 is the most frequent in Africa, Pakistan, East Asia, and north Indians (present study). The allele frequencies were found to be significantly different (*χ* ^2^ (trend) =10.981, *P* = 0.0009, *P*_*c*_ = 0.0063) between pediatric cataract cases and age-matched controls, where 26% of cases carried 13 “GATA” repeats as compared to 15% in controls (OR = 2.01, CI_95%_ = 1.2–3.3, *P* = 0.0054, *P*_*c*_ = 0.037) (Table 1). Allelic distribution was further analyzed according to longer (≥13 “GATA” repeats or ≥(GATA)_13_) and shorter (<13 “GATA” repeats or <(GATA)_13_) allele in cases and controls. The presence of a single or double copy of allele 6 (≥13 “GATA” repeats or at least one copy of longer allele) raised the risk of cataract further (OR = 2.11, CI_95%_ = 1.18–3.80, *P* = 0.0123, *P*_*c*_ = 0.0246), Table 2. Adjustment for gender, revealed the frequency of the longer allele to be significantly different among the male subjects (0.76 versus 0.49, OR = 3.17, CI_95%_ = 1.43–7.00, *P* = 0.0043, *P*_*c*_ = 0.0086) while no such difference was observed among the female subjects (0.71 versus 0.67, OR = 1.19, CI_95%_ = 0.49–2.92, *P* = 0.7006), Table 2. This difference remained significant even after Bonferroni correction. Out of the 10% of patients reporting family history positive for cataract, 6 were heterozygous and 3 were homozygous for the longer allele. Additionally, genotyping of D2S2944 marker in genomic DNA samples obtained from either of the parents of 5 pediatric simplex cataract cases showed three parents homozygous and one was heterozygous for the longer allele. The frequency of the major “C” allele of rs2289917 differed significantly between cases and controls (92% versus 80%, OR=2.989, CI_95%_ = 1.26–7.11, *P* = 0.0132, data not shown). The LD estimation by SHEsis indicated that rs2289917 and D2S2944 were in low LD *D*′ = 0.273, *r* ^2^ = 0.011, *P* = 0.131. Haplotype analysis showed that the haplotype with “C” allele of rs2289917 and ≥(GATA)_13_ allele of D2S2944 was more prevalent in pediatric cataract cases versus controls (61% versus 34%, OR = 2.952, CI_95%_ = 1.595~5.463, Fisher's *P* = 0.000453), Table 3. 

**Figure 3:**
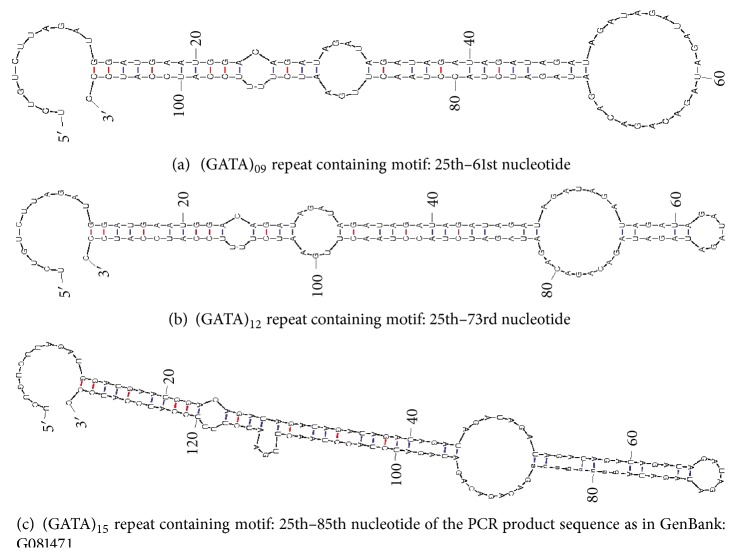
Structure analysis of transcripts containing different number of tetra repeats of D2S2944 marker. MFOLD predicted stem-loop structure depending on the number of (GATA)_*n*_ repeats in the microsatellite sequence of D2S2944 (GenBank: G08147.1) present in the intron 10 of *SPAG16* gene. Energetically more favorable stem-loop structure are formed in presence of >(GATA)_13_ repeats.

Control cohort segregated on the basis of castes showed no differences in longer allele frequency, indicating absence of any effect of population structures. In a replication cohort from western India, that is, Shekhawati region of Rajasthan 16% of healthy adult subjects out of 107 individuals have 13 “GATA” repeats of D2S2944, which is similar to the observed control frequency in the present studied cohort. The study has a power of >80% at *P*_0_ = 0.56 (controls with longer allele) and relative risk of 2 (risk of disease associated with presence of longer allele) with the given sample size. No significant difference in allele frequency of longer allele exists between the various endo-phenotypes of cataract. The putative structures of partial intronic sequence of the *SPAG16* gene covering the microsatellite D2S2944 showed that the mRNA fragment had the potential to form stem loops in the microsatellite motif. The stem-loop structure was energetically more favorable when the number of (GATA)_*n*_ repeats were >13 (ΔG for (GATA)_09_: −19.90 kcal/mol; ΔG for (GATA)_12_: −21.40 kcal/mol; ΔG for (GATA)_15_: −22.60 kcal/mol), suggesting that intronic region of *SPAG16* may also regulate *SPAG16* mRNA through post-transcriptional events, Figure 3.

## 4. Discussion

About one-third to one-half of all bilateral pediatric cataracts has a genetic basis with Mendelian inheritance [23]. However, extensive screening for variations in known disease associated genes could not identify the molecular lesion in large fraction of the families with inherited cataract in independent studies done in Australians, Indians, and Europeans [23–25]. Plausibly unidentified genes may be a more significant cause of cataracts than previously thought. The present study assessed the effect of D2S2944 marker on susceptibility for cataract in pediatric subjects. A significant difference in allele frequencies of longer (≥13 “GATA” repeats) and shorter alleles of D2S2944 was observed among the cases verses controls. Beem et al, have shown dominance of (GATA)_14_ tetra repeat allele of D2S2944 and its association with depressive individuals which are known to be at a higher risk for developing cataract [26, 27]. Our results are consistent with the findings of Kapoor et al. (2010) and Maher et al.(2010) who have again confirmed that allele 7 of D2S2944 marker is associated with major depressive disorder and mood disorders, respectively, in a sex-specific manner [28, 29].

Evidences from academic literature have indicated that expandable repeats, due to their unusual structural features, disrupt cellular replication, repair, and recombination machineries altering gene expression in human cells leading to disease. Many of these debilitating diseases are caused by repeat expansions in the non-coding regions of their resident genes [30]. Analysis of contig of chromosome 2 shows that the intragenic marker D2S2944 lies 225kb downstream of exon 10 of *SPAG16L* transcript, where an “untranslated exon” ahead of the first coding exon of SPAG16S (exon 11 of *SPAG16L*) has been reported [16]: Figure 1(b). Studies have shown that microsatellite motifs in the UTR form structural elements (stem loops) and contribute to mRNA regulation [14]. Our prediction using the MFOLD program not only showed stem-loop structures formed by sequences containing the D2S2944 microsatellite motifs but also favorable free energy level of sequence with >(GATA)_13 _repeats as compared with sequence with <(GATA)_13 _repeats. We here propose that the stabilized stem-loop structure due to microsatellite repeat expansion could affect the splicing mechanism normally taking care of the formation of *SPAG16S *and *SPAG16L* transcript. Consequently, affecting the expression of neighboring genes involved in maintaining lens transparency during developmental stages. In fact, Zhang et al., (2007) either could not detect the truncated SPAG16 protein in the western blots of sperm extracts from the human subjects carrying the SPAG16 heterozygous mutation (mutations that disrupt the expression of both SPAG16L and SPAG16S), reinforcing mRNAs transcripts instability [16]. 

Zhang et al. (2004) earlier reported mark impairment in spermatogenesis in mouse with the heterozygous mutation present in exon 11 of the *Spag16* gene which affected the expression of both L and S isoform of SPAG16 protein [31]. However, in humans haploinsufficiency of SPAG16L/SPAG16S does not impair male fertility [16]. This lends support to our observation where 60% of parents of simplex pediatric cataract cases were homozygous for the risk allele. Thus bracing the fact that the mutation/s in the *SPAG16* gene does not have a reproductive disadvantage but rather may have a profound effect on cell viability. The loss of L isoform of SPAG is responsible for instability of central apparatus components of the sperm. While the loss of SPAG16S transcript in addition to affecting Spag16L mRNA expression, affects postmeiotic germ cell viability [15, 16]. Further the distinct colocalization of SPAG16S with SC35 in nuclear speckles (nonnucleolar domains containing splicing factors as well as TFs, RNA processing units, and structural scaffold proteins) linked to the development of a cell-type specific genomic organization explains the S isoform's indispensable role in early developmental process [15]. 

It is noteworthy that the *CRYGB *gene is present 52, 77,275 bases upstream of D2S2944 marker (NCBI build 37.1) and has been previously reported to show strong gender differences in expression levels as well [32]. Both markers that is, rs2289917 and D2S2944 fall in high LD blocks according to HapMap (*D*′ = 1, LOD > 2). So it is possible that combined effect of risk allele of D2S2944, and rs2289917 is because there may be low LD (as shown by our *in silico* analysis) between some neighboring SNP and D2S2944 which explicate the association. We also observed novel sequence variations in the promoter region of *CRYGB *gene of pediatric cataract patients, which affected the putative TF binding sites in *in silico* analysis [4]. Being a WD-repeat protein, SPAG16 is known to interact dynamically and reversibly with TFs. Loss of SPAG16 protein and a promoter polymorphism affecting the TF binding in lens transparency maintaining gene/s thus together (as shown by our results) can either be a founder or disseminating event in early lens opacity progression. Recently, SPAG16 was found to be ubiquitously expressed in humans and has a testis associated alternative splice variant which has oncogenic properties [33]. Also expressed sequence tag profile of *SPAG16* gene at NCBI indicates the highest restricted pool expression in fetus in developmental stages. It is thus plausible that SPAG16 affect the expression of the neighboring genes which could be involved in maintaining lens transparency that is, *CRYGB* gene. In conclusion, to the best of our knowledge, this is the first example of a testis specific gene conferring the ability to regulate lens transparency in developmental stages. However a larger study is warranted for elucidating the molecular mechanism/s underlying the relationship between *SPAG16* gene and cataract.

## Figures and Tables

**Table 1:** Allele and genotype frequencies of D2S2944 microsatellite repeat in pediatric cataract cases and controls.

PCR Pdt Size (bp)	(GATA)_*n*_ repeats	Allele	Cases *n* = 97	Control *n* = 110
Allele frequency, 2*n* (%)				

100	08	1	0 (00)	0 (00)
104	09	2	0 (00)	4 (02)
108	10	3	8 (03)	12 (05)
112	11	4	19 (10)^a^	42 (19)^a^
116	12	5	48 (25)	69 (31)
120	13	6	50 (26)^b^	32 (15)^b^
124	14	7	56 (29)	46 (21)
128	15	8	13 (07)	15 (07)
132	16	9	0 (00)	00 (00)

Genotype frequency, *n* (%)				

<120	<13	6	26 (27)	48 (44)
<120/≥120	<13/≥13	<6/≥6	24 (25)	31 (28)
≥120	≥13	≥6	47 (48)	31 (28)

Allele and genotype distribution in cases versus controls, respectively: *χ* ^2^ (trend) = 10.981, *P* = 0.0009, *P*_*c*_ = 0.0063; *χ* ^2^ (trend) = 9.669, *P* = 0.0019, *P*_*c*_ = 0.0133. ^a^OR = 0.45, CI_95%_ = 0.25–0.81, *P* = 0.0079, *P*_*c*_ = 0.0553; ^b^OR = 2.01, CI_95%_ = 1.23–3.33, *P* = 0.0054, *P*_*c*_ = 0.0378. Note—<13 includes all homozygotes with both alleles less than 120 bp PCR pdt length, while <13/>13 includes heterozygotes with at least one 120 bp PCR pdt length allele and ≥13: homozygotes with both alleles more than 120 bp PCR pdt length. Pdt = product.

**Table 2:** Presence of at least one longer allele (≥13 GATA repeats) of D2S2944 (dominant model): pediatric cataract cases versus control subjects.

Number of (GATA)_*n*_ repeats	Longer allele of D2S2944	Cases	Controls	Males *n* (%)		Females *n* (%)	
		*n* (%)	*n* (%)	Cases	Controls	Cases	Controls
≥13	Present	71^a^ (73.2)	62^a^ (56.4)	40^b^ (75.5)	32^b^ (49.2)	31^c^ (70.5)	30^c^ (66.7)
<13	Absent	26 (26.8)	48 (43.6)	13 (24.5)	33 (50.8)	13 (29.5)	15 (33.3)

^a^OR = 2.11, CI_95%_ = 1.18–3.80, *P* = 0.0123, *P*_*c*_ = 0.0246.

^b^OR = 3.17, CI_95%_ = 1.43–7.00, *P* = 0.0043, *P*_*c*_ = 0.0086.

^c^OR = 1.19, CI_95%_ = 0.49–2.92, *P* = 0.7006*.*

**Table 3:** SHEsis predictions of frequencies of rs2289917-D2S2944 haplotypes in Indian pediatric cataract cases and control subjects.

Haplotype rs2289917-D2S2944	Cases 2*n* (%)	Controls 2*n* (%)	Fisher's *P *	Odds ratio [CI_95%_]
C—<(GATA)_13_	47 (33.6)	29 (45.3)	0.111665	0.615 [0.337~1.122]
C—≥(GATA)_13_	85 (60.7)	22 (34.4)	0.000453	2.952 [1.595~5.463]
T—<(GATA)_13_	05 (03.6)	07 (10.9)	0.038958	0.301 [0.091~0.996]
T—≥(GATA)_13_	03 (02.1)	06 (09.4)	0.014189	0.200 [0.049~0.818]

Global *χ* ^2^ is 17.23, df = 3, Fisher's *P* value is 0.000642.

## Confilict of Interests

There is no conflict of interests among any of the authors of the paper being submitted.
